# Chaperones as Suppressors of Protein Misfolded Oligomer Toxicity

**DOI:** 10.3389/fnmol.2017.00098

**Published:** 2017-04-05

**Authors:** Benedetta Mannini, Fabrizio Chiti

**Affiliations:** ^1^Department of Chemistry, University of CambridgeCambridge, UK; ^2^Section of Biochemistry, Department of Experimental and Clinical Biomedical Sciences, University of FlorenceFlorence, Italy

**Keywords:** proteostasis, protein misfolding diseases, clustering of aggregates, oligomer toxicity, aggresomes, inclusion bodies, amyloid fibrils, neurodegeneration

## Abstract

Chaperones have long been recognized to play well defined functions such as to: (i) assist protein folding and promote formation and maintenance of multisubunit complexes; (ii) mediate protein degradation; (iii) inhibit protein aggregation; and (iv) promote disassembly of undesired aberrant protein aggregates. In addition to these well-established functions, it is increasingly clear that chaperones can also interact with aberrant protein aggregates, such as pre-fibrillar oligomers and fibrils, and inhibit their toxicity commonly associated with neurodegenerative diseases without promoting their disassembly. In particular, the evidence collected so far in different labs, exploiting different experimental approaches and using different chaperones and client aggregated proteins, indicates the existence of two distinct mechanisms of action mediated by the chaperones to neutralize the toxicity of aberrant proteins oligomers: (i) direct binding of the chaperones to the hydrophobic patches exposed on the oligomer/fibril surface, with resulting shielding or masking of the moieties responsible for the aberrant interactions with cellular targets; (ii) chaperone-mediated conversion of aberrant protein aggregates into large and more innocuous species, resulting in a decrease of their surface-to-volume ratio and diffusibility and in deposits more easily manageable by clearance mechanisms, such as autophagy. In this review article we will describe the *in vitro* and *in vivo* evidence supporting both mechanisms and how this results in a suppression of the detrimental effects caused by protein misfolded aggregates.

## Introduction

The various proteins that constitute the human proteome are functional if they fold correctly, remain soluble, can be trafficked properly, form functional complexes and perform their task correctly. These abilities rely on the existence of a proteostasis network and on its proper running. The proteostasis network is constituted by the translational machinery, a large body of molecular chaperones and co-chaperones, the autophagy/lysosome system (ALS) and the ubiquitin/proteasome system (UPS). Molecular chaperones and co-chaperones can be grouped in distinct families, including ribosome-binding chaperones, Hsp40s, Hsp70s, chaperonins, Hsp90s, Hsp100, prefoldins, small heat shock proteins (sHsps) and TPR-domain containing co-chaperones. It has been estimated that this large body of proteins amounts to *ca*. 330 distinct polypeptide chains in the human proteome (Brehme et al., 2014).

Molecular chaperones have long been recognized to: (i) assist protein folding from unfolded or partially folded states and promote formation and maintenance of multisubunit complexes; (ii) mediate protein degradation via the UPS or ALS systems; (iii) inhibit protein aggregation by binding to fully or partially unfolded states; and (iv) promote disassembly of undesired aberrant protein aggregates (Kaushik and Cuervo, 2012; Labbadia and Morimoto, 2015; Mogk et al., 2015; Balchin et al., 2016). Although this last function has long been known to involve only chaperones of the ClpB and Hsp104 families present in bacteria, protozoa, plants and fungi (Mogk et al., 2015), it has recently been discovered in higher eukaryotes as well (Nillegoda and Bukau, 2015; Nillegoda et al., 2015).

However, in addition to these four well established functions, evidence is mounting that molecular chaperones can also interact with protein aggregates (macromolecular species resulting from the aberrant self-assembly of proteins) and inhibit their toxicity without promoting their disassembly. In this review we will show the evidence in this direction, showing how molecular chaperones can interact with both protein oligomers and amyloid fibrils, that represent the small species forming at the beginning of the aggregation process and the fibrillar end-product of this phenomenon, respectively. We will describe the mechanism through which molecular chaperones suppress the detrimental effects of such aggregates in the absence of their disaggregation or clearance.

## Chaperones Bind to Protein Oligomers

An increasing number of reports has shown the ability of molecular chaperones to interact with oligomeric species that form early during the aggregation of proteins. The extracellular chaperone clusterin has been observed, through Thioflavin T (ThT) kinetics and dot blot assays, to bind to prefibrillar species formed by six different amyloidogenic proteins or peptides at a substoichiometric 1:10 molar ratio (Yerbury et al., 2007). Using transmission electron microscopy (TEM), ThT kinetics and size exclusion chromatography (SEC), Hsp104 was found to bind to Aβ_42_ oligomers and protofibrils, but also to small fibrils, and abolish their ability to convert into amyloid fibrils through their further addition to preformed fibrils, as well as to abrogate their capacity to seed the addition of monomers to the fibril surface, up to a Hsp104:Aβ_42_ stoichiometric ratio of 1:1000 (Arimon et al., 2008). By means of single-molecule fluorescence methods, clusterin (Narayan et al., 2011) and the sHsp αB-crystallin or HSPB5 (Narayan et al., 2012) were observed to form long-lived, stable complexes with Aβ_40_ oligomers at equimolar ratios. Such sequestration was found also in the case of αB-crystallin and SOD1 aggregates, using ThT kinetics and SEC coupled with SDS-PAGE, with αB-crystallin:SOD1 molar ratios of 1:100 and 1:1, respectively (Yerbury et al., 2013). A quantitative kinetic analysis and immunochemistry studies revealed that the chaperone DNAJB6, from the Hsp40 family, preferentially binds to oligomeric species of Aβ_42_ at low substoichiometric molar ratios (up to 1:200), preventing their growth into longer fibrils as well as the formation of new fibril nuclei (Månsson et al., 2014).

This ability of chaperones to bind to protein oligomers is not just aimed at interfering with the process of amyloid fibril formation, but has also the important effect to inhibit directly the toxicity of these aberrant species. In fact, oligomers are characterized by physicochemical properties that make them harmful to cells (Campioni et al., 2010; Olzscha et al., 2011; Bemporad and Chiti, 2012). Among the structural determinants of toxicity identified so far, the small size and the high extent of hydrophobic surface of the oligomers have been recognized to play an important role in causing cellular dysfunction (Bolognesi et al., 2010; Cizas et al., 2010; Mannini et al., 2014). It is just on these structural determinants that molecular chaperones seem to act with the goal of counteracting the damages mediated by the oligomers: in fact chaperones increase the size of the oligomers and mask hydrophobic patches exposed on their surface. Such evidence will be described in the next two sections.

## Chaperones Induce Clustering of the Oligomers and Inhibit Their Toxicity

In an early report, the ability of clusterin to inhibit the toxicity of preformed oligomers of Aβ_42_ and of the SH3 domain of phosphatidylinositol 3-kinase (PI3-SH3) was observed on human neuroblastoma SH-SY5Y cells at 1:10 clusterin:substrate ratio (Yerbury et al., 2007). Although the mechanism of action of the chaperone against the aggregates was not investigated in detail, the authors reported a sedimentation assay in which the formation of high molecular weight Aβ_42_/clusterin complexes were observed (Yerbury et al., 2007).

Later on, Hsp27 (HSPB1) was found to increase the size of preformed Aβ_42_ oligomers, making them unable to exert their toxicity on N2a mouse neuroblastoma cell cultures (Ojha et al., 2011). In particular, atomic force microscopy (AFM), TEM and light scattering showed that the incubation of a substoichiometric concentration of Hsp27 with preformed Aβ_42_ oligomers (1:5) *in vitro* generates larger aggregates. In these larger species Hsp27 co-precipitated with the Aβ_42_ oligomers, without affecting significantly the structure of the oligomers, as shown by unaltered ThT binding and far-UV circular dichroism (CD) spectra. Interestingly, even the ability to bind to 8-anilinonaphthalene-1-sulfonic acid (ANS) was unalterd, indicating that the extent of the hydrophobic surface exposure does not change after the incubation with the chaperone.

In another study, five different chaperones, namely, αB-crystallin, Hsp70 (HSPA1A), clusterin, α_2_-macroglobulin and haptoglobin were able to suppress the toxicity of oligomers formed by three different proteins, the Aβ_42_ peptide, the islet amyloid polypeptide (IAPP), and the model protein HypF-N at a substoichiometric concentration (up to 1:1000) on SH-SY5Y cells (Mannini et al., 2012). Several methods of investigation applied to the HypF-N system, such as AFM, confocal microscopy coupled to immunostaining, centrifugation assays and intrinsic fluorescence, showed that large clusters of HypF-N oligomers are formed following the incubation with substoichiometric concentrations of chaperones. In the resulting large aggregates, chaperone molecules are trapped inside the large aggregates. Again, the chaperone-mediated conversion of the small oligomers into bigger nontoxic aggregates occurs without a remodeling of the structure of the oligomers, as assessed by Fourier-transform infrared spectroscopy and pyrene labeling (Mannini et al., 2012).

Recently, it was found that high amounts of insoluble aggregates containing chaperones, in particular the sHsps, accumulate in the long-lived daf-2 mutants of the nematode *Caenorhabditis elegans* during aging, and that such amounts are higher compared to the wild-type controls that had a normal lifespan (Walther et al., 2015). It was suggested that the chaperones neutralize aberrant, potentially toxic, proteins and soluble oligomers by driving them into insoluble large aggregates and that this strategy enables to slow down the decline of the proteostasis network during normal aging and extend the lifespan of the mutant nematodes (Walther et al., 2015).

A similar mechanism was observed on various cell culture systems, such as CHO, N2a, NIH-3T3, PC12 and MEF, treated with NT219, an inhibitor of the insulin/IGF-1 signaling pathway (Moll et al., 2016). NT219 was found to enhance the aggregation of misfolded prion proteins and promote its deposition in intracellular inclusions such as the aggresomes. Although NT219 was also found to increase the concentrations of certain molecular chaperones, it also reduces proteasome activity and impairs autophagy, indicating that conversion of proteins into large aggresomes is a protective mechanism even in the absence of their immediate clearance (Moll et al., 2016).

## Chaperones Shield the Hydrophobic Moieties of the Oligomers and Inhibit Their Toxicity

The chaperone-induced clustering of aberrant protein oligomers is not the only mechanism through which these important protein molecules protect against oligomer toxicity. Evidence has been shown, using surface plasmon resonance (SPR), on the ability of clusterin to bind to toxic Aβ_42_ oligomers at substoichiometric ratio (1:1000) and shield their reactive hydrophobic patches on their surface (Beeg et al., 2016). The pre-incubation of these aggregates with clusterin also reverted their ability to reduce the pharyngeal mobility in *C. elegans* nematodes (Beeg et al., 2016). In another study, Hsp70 was modified to be released in the extracellular space in order to address its protective activity against Aβ_42_ in *Drosophila melanogaster* models (Fernandez-Funez et al., 2016). The secreted form, called secHsp70, suppressed Aβ_42_ toxicity, as deduced by decreased eye degeneration, reduced neuronal death, structural integrity of adult neurons, suppression of locomotor neuron dysfunction, and lifespan extension. An assay based on luciferase-derived luminescence showed that secHsp70 stabilizes Aβ_42_ oligomeric species and masks their neurotoxic epitopes, thus promoting the accumulation of nontoxic aggregates (Fernandez-Funez et al., 2016). This activity was carried out in the absence of ATP, indicating therefore that secHsp70 exploits its holdase function to interact with the oligomers in the absence of detectable clustering (Fernandez-Funez et al., 2016). It is interesting to note that other Hsps that were found able to interact with aggregates, such as Hsp104 (Arimon et al., 2008; Castellano et al., 2015), Hsp70 (Mannini et al., 2012) and Ssa1p (Xu L. et al., 2013), exert the capacity to bind to the aggregates without consumption of ATP.

Stabilization of oligomeric Aβ_42_ has also been observed in the presence of human prefoldin (hPFD) at substoichiometric molar ratio (up to 1:500) using western blot and TEM. Viability assays on cultured PC12 cells or primary cortical neurons from embryonic mice show that the Aβ_42_/hPFD complexes are less toxic than complexes of similar size obtained by incubating Aβ_42_ oligomers with archaeal prefoldin (PhPFD). The different biological activity was attributed to the higher hydrophobic exposure and β-sheet content of Aβ_42_/PhPFD complexes, as assessed by ANS and ThT binding (Sörgjerd et al., 2013).

These observations highlight the existence of different mechanisms of action mediated by the chaperones against the toxicity of the oligomers, that is “binding followed by clustering” and “binding causing hydrophobic shielding in the absence of clustering”. Both mechanisms have been indeed observed on a recent report in which the effect of the chaperones αB-crystallin and clusterin, and an engineered monomeric variant of transthyretin known to have a chaperone-like activity, was investigated over a wide range of concentrations, both super- and sub-stoichiometric relative to HypF-N toxic oligomers, ranging from 4:1 to 1:16 (Cappelli et al., 2016). AFM images and light scattering measurements showed that the chaperones increase the size of the aggregates to an extent that correlates with chaperone concentration, ranging from null to remarkable increase. Notably, the protective effect on N2a cells was observed at all chaperone concentrations, irrespective of the size increase. Measurements of ANS binding showed that in the large clusters the overall exposure of the hydrophobic surface does not change, whereas when the clustering promoted by the chaperones is negligible the ANS binding is reduced, indicating that the hydrophobicity on the surface is shielded by the chaperones.

## Chaperones Bind to Amyloid Fibrils

Prefibrillar oligomers are not the only molecular target of molecular chaperones. An increasing body of reports also describe the ability of these guardian proteins to bind to mature fibrils. In this regard, the sHsp αB-crystallin has been extensively studied because it colocalizes with Aβ plaques (Shinohara et al., 1993) and Lewy bodies (Wakabayashi et al., 2007), that are the neuropathological hallmarks of Alzheimer’s disease and Parkinson’s disease, respectively. Indeed, this chaperone has been found to bind to fibrils from Aβ_40_ (Raman et al., 2005), Aβ_42_ and the Arctic variant E22G Aβ_42_ (Shammas et al., 2011), and α-synuclein (Waudby et al., 2010). The binding prevents fibril growth of Aβ_40_, as revealed by ThT binding assays, total reflection fluorescence microscopy and CD measurements (Raman et al., 2005). A strong inhibition of fibril growth was also demostrated for Aβ_42_ (Shammas et al., 2011) and α-synuclein (Waudby et al., 2010), by measurements of seeded fibril elongation kinetics, both in solution and on the surface of a quartz crystal microbalance (QCM). Immunoelectron microscopy images showed that αB-crystallin binds along the entire lenght and ends of the Aβ_42_ (Shammas et al., 2011) and α-synuclein fibrils (Waudby et al., 2010). Although cell toxicity measurements were not reported directly, it was hypothesized that the binding of αB-crystallin to fibrils may reduce their toxicity by shielding the exposed hydrophobic residues and by preventing the generation of new oligomers that occurs on the fibril surface due to secondary nucleation (Waudby et al., 2010).

The ability of αB-crystallin to bind to fibrillar species and inhibit their growth has also been observed with many other non-neuronal amyloid fibril systems, such as insulin fibrils at low pH (Knowles et al., 2007), β_2_-microglobulin (β_2_-m) fibrils at low pH (Raman et al., 2005) and apolipoprotein C-II (apoC-II) fibrils at neutral pH (Binger et al., 2013). Interestingly, by exploiting the property of β_2_-m fibrils to depolymerize when shifted to neutral pH, it was found that αB-crystallin retards fibril depolymerization (Raman et al., 2005). A chaperone-induced stabilization effect was also observed in the case of apoC-II fibrils, when their fragmentation promoted by dilution was inhibited in the presence of the chaperone (Binger et al., 2013). In the same report, αB-crystallin was also found, using TEM images and sedimentation assays, to induce the formation of large fibrillar tangles. Although the authors did not provide direct experimental evidence, they argued that these stabilized clumped inclusions represent a protective strategy because they are unable to release cytotoxic oligomers and to promote events of secondary nucleation (Binger et al., 2013).

A direct proof of the beneficial effect of the binding of chaperones to fibrils was obtained for the human Brichos domain (Cohen et al., 2015). This chaperone binds to the surface of Aβ_42_ fibrils, where the formation of oligomeric intermediates is catalyzed, and therefore minimizes the formation of toxic species, as demonstrated by several techniques, such as ThT kinetic analysis, TEM coupled with immunogold labeling, SPR and SEC in cojunction with immunoblot. Electrophysiology experiments in living mouse brain tissue, as well as cell viability measurements based on the MTS assay and the capsase-3 activity quantification on SH-SY5Y cultured cell lines, verified that this mechanism effectively suppresses the oligomer-mediated damage.

## The Ability of Other Non-Chaperone Proteins to Bind to Protein Aggregates and Inhibit Their Toxicity

Other proteins that are generally not classified as chaperones have been recognized to bind to aggregates and suppress their toxic effects, thus acting as officially recognized molecular chaperones. Soluble collagen VI was found to rescue mice neocortical/hippocampal neurons from the toxicity mediated by Aβ_42_ oligomers by altering the interaction of the oligomers with neurons (Cheng et al., 2009). Indeed, immunostained confocal microscopy images showed that collagen VI prevents the association of Aβ_42_ oligomers with the surface of cultured neurons and was found to colocalize with Aβ_42_ into large deposits in the extracellular space, with the latter finding being confirmed by AFM. This mechanism of sequestration was found to result in a lower amount of soluble toxic oligomers in the extracellular space, with lack of binding to the neuron surface and protection from the damage (Cheng et al., 2009).

This protective strategy has also been observed, by means of immunolabeled confocal microscopy images, for the complement protein C1q, shown to prevent the association of fibrillar Aβ_42_ to cultured mouse primary cortical neurons and to increase the size of Aβ_42_ oligomeric species (Benoit et al., 2013). In particular, the accumulation of the oligomers into large deposits in the extracellular space impedes their internalization in the neurons, as demonstrated by the lower colocalization between the oligomers with a lysosomial marker in samples treated with C1q (Benoit et al., 2013).

Interesting is the case of transthyretin, which has been recently found to possess a generic ability to deal with protein misfolded oligomers (Li et al., 2011, 2013; Cascella et al., 2013). When pre-incubated with two different oligomeric species formed by the Aβ_42_ peptide and the HypF-N protein, human tetrameric transthyretin (hTTR) and its engineered monomeric variant (M-TTR) effectively suppress their toxicity on SH-SY5Y cell cultures, again promoting the clusterization of the oligomers into larger species, as shown by AFM and immunostained confocal microscopy, in the absence of their structural reorganization, as shown by ThT fluorescence and the patterns of the pyrene spectra (Cascella et al., 2013).

The maltose binding protein (MBP) from *Escherichia coli* has been shown to induce the formation of bigger clusters of preformed Aβ_42_ oligomers, visible in TEM images, which have a reduced perniciouness to SH-SY5Y cell cultures (Sharoar et al., 2013). These assamblies were found to display a lower level of ThT binding and β-sheet content in the CD spectra, indicating in this case a change in the secondary structure induced by MBP. Finally, the protein α_s1_-casein from bovine milk was found to inhibit fibril generation of Aβ_40_ by redirecting the process towards the formation of amorphous aggregates (Carrotta et al., 2012). AFM images and data obtained with CD spectroscopy and ThT binding assays showed that α_s1_-casein affects the formation of the oligomers and the growth of the protofibrils. The aggregates formed in its presence have a lower content in fibrillar structure and a large globular appearance (Carrotta et al., 2012).

Following all this experimental evidence it is clear that many proteins cooperate with molecular chaperones in defending the cells from the insults caused by aberrant protein oligomers and that a widespread proteostasis control exists, with a multiplicity of guardians *in vivo*.

## Assembly of Protein Oligomers and Fibrils into Large Aggregates *In Vivo*

The neutralization of protein misfolded oligomers and fibrils following their binding to molecular chaperones and subsequent clustering into larger aggregates observed *in vitro* has also been found *in vivo*. It has been shown that in all living organisms, from bacteria to high eukaryotes, the aggregates formed intracellularly are assembled together in one or a limited number of inclusions (Villaverde and Carrió, 2003; Hyttinen et al., 2014; Miller et al., 2015), which are termed aggresomes in mammalian cells and are in close proximity to the centrosome (Johnston et al., 1998; Hyttinen et al., 2014).

The formation of the aggresome is thought to be a protective process able to sequester harmful aggregates and to act as a storage center for eventual degadation via authophagy (Villaverde and Carrió, 2003; Hyttinen et al., 2014; Miller et al., 2015). The assembly of small aggregates into the large aggresome is not mediated by a single chaperone, as observed in simplified experiments *in vitro*, but is a finely regulated process mediated by a complex machinery: misfolded aggregates are polyubiquitinated and associate with the microtbule-associated protein dynein, which transports them to the microtubule organizing center (MTOC) to merge them into the growing aggresome (Hyttinen et al., 2014). The association of the polyubiquitinated aggregate to the microtubule/dynein complex is mediated by chaperones of the Hsp70 family, the Hsp70 co-chaperone Bcl-2-associated athanogene 3 (BAG-3) and the protein 14-3-3 which has binding sites for both BAG-3 and dynein (Xu Z. et al., 2013; Jia et al., 2014). It can also be mediated by histone deacetylase 6 (HDAC6), which has binding sites for both ubiquitin and dynein (Kawaguchi et al., 2003; Ouyang et al., 2012). Importantly, however, the outcome is similar to that observed *in vitro*, that is neutralization of diffusible and potentially harmful oligomers into an innocuous and easily manageable large aggregate, which will later be degraded via autophagy.

Formation of large inclusion bodies has also been widely studied in yeasts, where three distinct inclusion bodies have been observed, namely the cytosolic quality control bodies (Q-bodies or cytoQ), the intranuclear quality control compartment (INQ, previously termed JUNQ) and the insoluble protein deposit (iPOD) forming close to the vacuole (Miller et al., 2015). Although the molecular mechanisms underlying formation of such inclusions are still largely unclear, it has been shown that cytoQ and INQ form from the fusion of smaller aggregates and that their formation is mediated by chaperones such as the small heat shock protein Hsp42 and the heat shock protein Btn2, respectively (Miller et al., 2015).

All the results that have shown the ability of molecular chaperones to interact with protein oligomers and neutralize their deleterious effects have mainly been obtained in cultured cell models. As described above, there is evidence that chaperones also induce formation of large aggregates *in vivo*, but the molecular mechanism by which this occurs and how such a complex tissue as the human brain benefits from this possibly protective process awaits specific experimental studies.

## Conclusions

**Figure 1:**
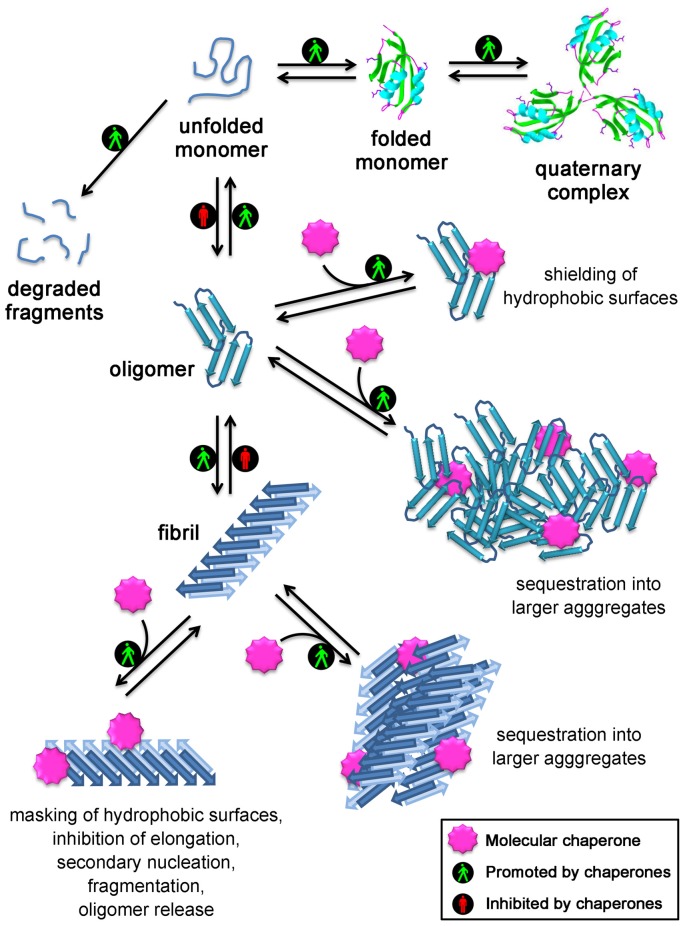
**Mechanisms used by molecular chaperones to maintain proteins in their soluble states and reduce the toxicity of protein aggregates.** Chaperones assist protein folding, promote formation and maintenance of multisubunit complexes, mediate protein degradation, inhibit protein aggregation and promote disassembly of undesired protein aggregates. In addition, several strategies are employed by molecular chaperones to reduce the toxicity of protein aggregates: they act on small soluble oligomers by shielding their hydrophobic patches or by sequestering them into larger aggregates; they also promote the clustering of the fibrils, inhibit their elongation, the generation of oligomers through secondary nucleation occurring on the fibril surface, their fragmentation/oligomer release, and mask the reactive hydrophobic residues exposed on the fibril surface.

Overall, all the experimental evidence collected so far from both *in vitro* and *in vivo* studies indicate that chaperones do not just maintain proteins in their soluble native states, as thought until 5 years ago, but also directly bind to protein oligomers and fibrils and neutralize their deleterious effects. This may occur through: (i) the direct binding to the hydrophobic patches exposed on the oligomer/fibril surface, which are responsible for the aberrant interactions with a number of targets in the cell and on the cell membrane; or (ii) their conversion into large and more innocuous species, which appear to minimize their surface-to-volume ratio, their diffusibility and to be more easily manageable by clearance mechanisms, such as autophagy. A schematic representation of the mechanisms used by molecurar chaperones to maintain protein homeostasis is shown in Figure 1. In the light of the fact that the formation of large aggregates in the cells has a protective role, it is conceivable that the large size and spatially circumscribed nature of the histopathological signatures of various neurodegenerative diseases, such as the amyloid plaques and neurofibrillarly tangles in Alzheimer’s diseases, the Lewy bodies in Parkinson’s disease, the round and skein inclusions in amyotrophic lateral sclerosis, represent an *extrema ratio* of the cells to limit the damages of these undesired oligomers, the choice by the cell of the lesser of two evils: the small reactive oligomers and the large inert deposits, that become unable to be cleared with aging or disease progression.

## Author Contributions

FC and BM designed the work, performed literature search, wrote the manuscript and critically revised it.

## Conflict of Interest Statement

The authors declare that the research was conducted in the absence of any commercial or financial relationships that could be construed as a potential conflict of interest.
